# WHAT IS THE KNOWLEDGE OF ELEMENTARY SCHOOL TEACHERS ABOUT SCOLIOSIS?

**DOI:** 10.1590/1413-785220233101e254450

**Published:** 2023-02-20

**Authors:** ANDERSON ALVES DIAS, MARCOS VINICIUS LAZARINI DA CUNHA, LEONARDO FRANCO PINHEIRO GAIA, NÍCOLAS SANTOS DE OLIVEIRA, ANDRÉA LICRE PESSINA GASPARINI, ISABEL APARECIDA PORCATTI DE WALSH, DERNIVAL BERTONCELLO

**Affiliations:** 1 Hospital Lifecenter, Orthopedics Service, Belo Horizonte, MG, Brazil.; 2 Universidade Federal do Triângulo Mineiro, Institute of Health Sciences, Uberaba, MG, Brazil.; 3 Universidade de São Paulo, Department of Orthopedics and Traumatology, São Paulo, SP, Brazil.; 4 Universidade de Ribeirão Preto, Department of Physical Therapy, Ribeirão Preto, SP, Brazil.; 5 Universidade Federal de São Carlos, Department of Physical Therapy, São Carlos, SP, Brazil.

**Keywords:** Scoliosis, School Teachers, Adolescent, Prevalence, Escoliose, Professores Escolares, Adolescente, Prevalência

## Abstract

**Objective:**

To evaluate the knowledge about scoliosis in teachers of municipal public schools.

**Methods:**

In total, 126 professionals were interviewed using a standard questionnaire containing issues related to scoliosis.

**Results:**

31% of interviewees did not know what scoliosis is. Of those who knew 89.65% were partially correct about the definition. Of those who claimed to know how the scoliosis diagnosis is made, only 25.58% were completely correct. When questioned about the Adams test, 84.9% did not know it. Among the interviewees, 57.9% answered that it is impossible to identify scoliosis by a simple examination of their students and, off these, 86.3% stated the lack of knowledge about the subject; and 92.1% considered that training for the diagnosis and early identification of scoliosis in students.

**Conclusion:**

This study holds social impact since the interviewed teachers were not knowledgeable about the subject and had difficulty in providing a definition of the condition and in how to proceed with the investigation. Continuous education activities and the inclusion of this subject on the curricula of teacher education programs would improve the early diagnosis and treatment of scoliosis, with high success rates. ***Level of Evidence IV, Economic and Decision Analyses.***

## RESUMO

**Objetivo:**

Avaliar o conhecimento de professores de escola municipal sobre escoliose.

**Métodos:**

Foram entrevistados 126 profissionais por meio de formulário online padronizado contendo perguntas relativas à escoliose.

**Resultados:**

Dos entrevistados, 31% não sabiam o que é escoliose. Dos que sabiam, 89,65% estavam parcialmente corretos quanto à definição. Dos professores que responderam saber como era feito o diagnóstico de escoliose, apenas 25,58% estavam totalmente corretos. Quando questionados sobre o teste de Adams, 84,9% não sabiam do que se tratava. Dos entrevistados, 57,9% disseram que não é possível identificar escoliose por meio de um exame simples de seus alunos em sala de aula e, destes, 86,3% alegaram falta de conhecimento sobre o assunto; e 92,1% consideraram que é importante haver uma capacitação sobre o diagnóstico de escoliose para identificação precoce nos alunos.

**Conclusão:**

Este estudo tem impacto social, pois os professores entrevistados não apresentavam domínio sobre o assunto, demonstrando dificuldade em definir a condição e como proceder com a investigação. A realização de capacitações e a inclusão dessas questões no currículo profissional aumentaria o diagnóstico precoce de escoliose nas escolas, possibilitando o tratamento precoce e maiores chances de sucesso terapêutico. **Nível de Evidência IV, Análise Econômica e de Decisão.**

## INTRODUCTION

Adolescent Idiopathic Scoliosis (AIS) is an anatomical and structural alteration of the spine with a lateral curve in the coronal plane, often with a rotational component, measured above 10° using the Cobb method.^1^ AIS affects people over 10 years of age and is more prevalent in women. The etiology is unclear and different causal factors are suggested, including neuromuscular or connective tissue changes, asymmetric growth of the trunk and limbs, changes in the sagittal configuration of the spine and hereditary and environmental factors, such as feeding.^2^

Studies about the prevalence of scoliosis in Brazil are restricted to isolated populations, but estimations show a worldwide prevalence of AIS ranging from 1% to 13%.^3^ The progression of the scoliosis curve is greater during the growth spurt phase, which occurs before skeletal maturity, so its detection in the school years is important. Despite this, the lay population still neglects the disease in its early stages, which can result in severe consequences in adulthood. Greater knowledge about the subject on behalf of elementary school teachers should improve the early detection of abnormal curvatures of the spine and lead to better treatment and prevent the evolution of scoliosis. However, studies on the previous knowledge of teachers of public or private schools regarding AIS are rare.

This study aimed to verify the knowledge about scoliosis of teachers of municipal schools in a medium-sized city.

## MATERIALS AND METHODS

With the help of the Municipal Education Department, 126 teachers from municipal public schools in Uberaba, Minas Gerais, Brazil, who met the following inclusion criteria were selected: being Brazilian and teaching classes for children aged 10 to 13 years.

This was a quantitative cross-sectional study, conducted with the application of an online questionnaire via the Google Forms platform^®^ from September to October 2020, containing 10 basic questions on scoliosis to verify the knowledge of the professionals. This study was approved by the Research Ethics Committee of the University of Uberaba (CAAE 20150919.3.0000.5145). All participants signed an Informed Consent Form.

The objective of the research was explained to the teachers in a previous e-mail and the interviewees were then invited to participate. The responses were archived on Google Drive^®^ for analysis.

The study included 126 municipal public school teachers working with children aged 10 to 13 years. The questionnaire was prepared by the authors and consisted of simple objective and subjective questions. The following subjects were questioned: (1) if teachers knew what scoliosis is, (2) if they could define scoliosis, (3) how it is diagnosed, (4) if they knew what the Adams test is, whether scoliosis can be identified by a simple examination in students in the classroom and if not, what would be the reason, (5) if the training of teachers to diagnose scoliosis early in their students is important, (6) if the teacher knew someone with scoliosis. The results were transcribed for analysis. No similar study was found in the literature to compare the data obtained.

The representativeness of the teacher population was achieved by defining the minimum sample size according to the statistical formula for a simple random sample, finite universe, 95% confidence level, and 5% tolerable sampling error. Thus, the sample of 126 teachers presented significant relevance with a 95% confidence level. All results were first tabulated in spreadsheets to verify the variables. The Chi-squared test was then applied.

## RESULTS

**Figure 1:**
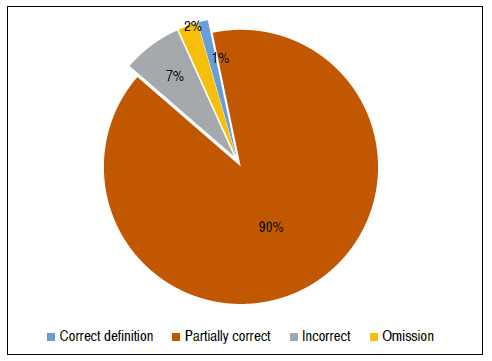
Percentage of accuracy regarding the definition of scoliosis.

In total, 126 responses were obtained from the questionnaires sent via Google Forms^®^. In our sample, 31% of respondents did not know what scoliosis is. Of the interviewees who knew (69%), 89.65% were partially correct when defining it. Only one teacher presented the correct definition, 6.9% provided wrong definitions, and two teachers did not answer (Figure 1).

When asked about how scoliosis would be diagnosed, 65.4% did not know how to answer. Of the teachers who answered that they knew, only 25.58% were totally correct, 69.78% were partially correct, and 1% incorrect. Still regarding the diagnosis when asked about what the Adams test was, 84.9% did not know (Table 1).

**Table 1:** Absolute numbers and percentage about the knowledge on the diagnosis of scoliosis.

Type of answer	Number of answers (%)
Correct	11 (25.58%)
Partially correct	30 (69.76%)
Incorrect	1 (2.33%)
Omission	1 (2.33%)

Teachers were asked if scoliosis can be identified by a simple examination in students in the classroom and 57.9% said no. Of these, 86.3% said that the difficulty would be due to the lack of knowledge on the subject; 92.1% consider that training on the diagnosis of scoliosis for early identification in students is important (Table 2).

**Table 2:** Justifications presented by teachers about the possibility of identifying scoliosis in students by a simple examination.

Justification for not identifying scoliosis	Number of answers (%)
Lack of knowledge	63 (86.3%)
Lack of time to perform it	3 (4.11%)
Does not understand it as a teacher's duty	5 (5.48%)
Other reasons	3 (4.11%)

**Figure 2:**
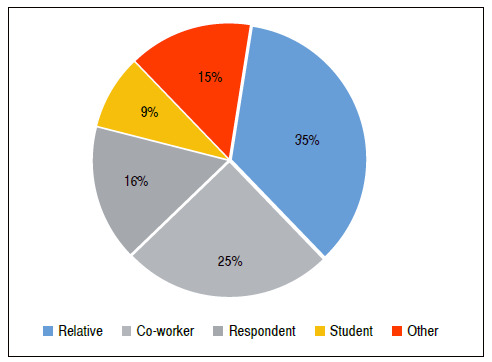
Percentage of teachers who know someone with scoliosis, whether a student, co-worker, relative, the respondent themselves, and other individuals.

Of the interviewees, 54% know someone with scoliosis, of which 60.3% are co-workers or relatives (Figure 2). Only 8.82% reported knowing a student with scoliosis. This study has a social impact, since interviewees lacked knowledge on scoliosis, had difficulty in defining and diagnosing it, and in how to examine students in the classroom.

## DISCUSSION

When scoliosis is detected early, the involvement of people close to adolescents is also needed. Knowledge about scoliosis, conceptual or about prevention and treatment, is crucial for early detection and better solvability.

The prevalence of scoliosis diagnosed with Cobb angle greater than 10 degrees in the general population is around 0.93% to 12%. However, some studies show a variation of 2% to 3% according to the literature review by SOSORT.^4^

AIS is usually present during pre-adolescence or adolescence, a period of growth spurt. Postural changes in adolescence should be observed because they are a predisposing factor, that is, they may be related to the onset of the disease.^5^ Suspected cases of scoliosis require appropriate physical examination, as it is a pathology with no other symptom but deformity at first.^6^

Given the need to plan early diagnosis, professionals other than health ones should hold knowledge about the disease to direct adolescents to the appropriate health care. School teachers are an important population that could fill such a detection role, or that of being further observers of adolescent health. However, when one observes the undergraduate curricula of school teacher courses, no classes on the early detection of structural diseases, such as scoliosis, are present, which could be considered a gap given the importance of this diagnosis in school-age students.

The prognosis for adolescents with scoliosis evaluated past their growth phase is more unfavorable, evolving into chronic pain, mechanical and respiratory restrictions and, in more severe cases, cor pulmonale.^7^ This meets the need presented above.

Studies show a 1% to 13% worldwide incidence rate for AIS. The incidence rate generally reported for the school student population is 0.5% to 3%. The prevalence rates of scoliosis in school screening vary by country; in Brazil, it ranges from 2% to 4% in adolescents aged 10 to 16 years.^4^ The literature describes cases in relation to the epidemiology of AIS, such one conducted in Belo Horizonte, MG, Brazil, which found scoliosis in 4.8% of the 358 students observed.^8^

A study conducted in Niterói, RJ, Brazil, evaluated 4,750 asymptomatic adolescents and showed a 1.03% prevalence for idiopathic scoliosis, with a curve from 11 to 20 degrees by the Cobb method. This study evaluated 418 adolescents and found 18 cases, for a 4.3% prevalence, being compatible with the literature.^9^ Another study in Maranhão, Brazil, evaluated 7,295 students and the AIS prevalence by gender was 7.3% 15.8% in boys and girls, respectively.^10^

Another study used the Adams test for screening and found a 48.4% prevalence of lateral postural changes in students aged 10 to 12 years, and 49.5% of lateral alterations in students aged 13 to 15 years. Another study, also using the plumb line, but with younger students (6 to 15 years of age), of both sexes, found a 38.88% prevalence of lateral alterations and 33.27% prevalence of anteroposterior alterations.^11^

According to data from the Brazilian Institute of Geography and Statistics (IBGE), from 2010, the schooling rate from 6 to 14 years of age in Uberaba, Minas Gerais corresponds to 97.7%. In total, 36,729 adolescents were enrolled in elementary school in 2018 (from 11 to 16 years old).^12^ Considering that the age of 11 to 13 years represents 50% of the students enrolled, an estimation shows 18,000 students in this age group. Observing that the prevalence of scoliosis in adolescents aged between 11 and 13 years is, on average, 3%^4^ and of the estimated 18,000 students enrolled in this age group, 540 students should have scoliosis in the schools of the municipality.

Early diagnosis enables the effective treatment, almost always without the need for surgery, which is both costly and risky.^13^ When the curve becomes structured, that is, after the growth spurt phase, clinical treatment options lose efficiency. The curvature can thus impact important postural changes, pain, and changes in the respiratory pattern.^14^

Because scoliosis is more common in school-age adolescents, early detection can be optimized by training elementary school teachers to perform basic diagnostic procedures, such as the Adams test.^15^ This test uses a noninvasive method and consists of detecting spinal deformity on the patient’s back when they perform anterior trunk flexion.^16^ Early detection can be done by people in adolescents’ daily living, such as family members or teachers, which would function as a screening prior to diagnosis and refer them for the evaluation of primary health care professionals. This action could significantly contribute to the early treatment of scoliosis.

Preventive measures aiming at ergonomic aspects are also needed to evaluate postural changes early and educate children about the appropriate postures when studying, carrying school objects, and practicing physical exercises, thus avoiding the impairment of the musculoskeletal system,^15^ and informing them about the importance of maintaining a good posture to avoid current and future postural problems.^1^

This study bears social impact, as the interviewed teachers’ knowledge on scoliosis was lacking and they had difficulty in defining it and how to conduct a prior examination of students in the classroom. The inclusion of this topic on the curricula of these professionals could improve the early diagnosis of scoliosis, leading to early treatment and higher success rates.

Thus, knowledge of these data is important for its use in public and private health systems, as well as a resource for other studies on adolescent idiopathic scoliosis, better guiding improvements to health policies with an interface to education.

## CONCLUSION

Elementary school teachers, especially those of adolescent years, are unaware of basic concepts about idiopathic scoliosis.
